# Devising Hyperthermia Dose of NIR-Irradiated Cs_0.33_WO_3_ Nanoparticles for HepG2 Hepatic Cancer Cells

**DOI:** 10.1186/s11671-021-03565-4

**Published:** 2021-06-26

**Authors:** Po-Sheng Hu, Hsiu-Jen Chou, Chi-An Chen, Po-Yi Wu, Kai-Hsien Hsiao, Yu-Min Kuo

**Affiliations:** 1 College of Photonics, National Yang Ming Chiao Tung University, Tainan City, 71150 Taiwan; 2 College of Photonics, National Chiao Tung University, Tainan City, 71150 Taiwan; 3 Department of Cell Biology and Anatomy, College of Medicine, National Cheng Kung University, Tainan City, 70101 Taiwan

**Keywords:** HepG2 cell, Cesium tungsten oxide nanoparticles, Hyperthermia dose, Photothermal trigger, Cytotoxicity

## Abstract

Hyperthermia is one of the most patient-friendly methods to cure cancer diseases owing to its noninvasiveness, minimally induced side-effects and toxicity, and easy implementation, prompting the development of novel therapeutic methods like photothermally triggering dose system. This research herein interrogates the variables of photothermal effects of Cs_0.33_WO_3_ nanoparticles (NPs), the duration of irradiation, optical power density and NP concentration, upon HepG2 liver cancer cell line in vitro, leading to the formulation of a near-infrared (NIR)-irradiated thermal dose. Expressly, the NPs with particulate feature sizes of 120 nm were synthesized through a series of oxidation–reduction (REDOX) reaction, thermal annealing and wet-grinding processes, and the subsequent characterization of physical, compositional, optical, photothermal properties were examined using dynamic light scattering (DLS), energy-dispersive X-ray spectroscopy (EDS), scanning and tunneling electron microscopies (SEM and TEM), X-ray diffraction (XRD) and visible-near-infrared (VIS–NIR) photospectroscopy. Cytotoxicity of the NPs and its irradiation parameters were obtained for the HepG2 cells. By incubating the cells with the NPs, the state of endocytosis was verified, and the dependence of cellular survival rate on the variable parameters of photothermal dose was determined while maintaining the medium temperature of the cell-containing culture dish at human body temperature around 36.5 °C.

**Supplementary Information:**

The online version contains supplementary material available at 10.1186/s11671-021-03565-4.

## Introduction

Globally, in the year of 2018 along, ferocious cancer diseases claimed about 10 million lives and have added an estimated 18 million new cases [1]. Thus far, although chemotherapy, radiation, surgical removal or tailored combination of these three account for an improvement of the 5-year survival rate slightly over 40% of treated cancer patients [2, 3], the toxic and deleterious nature of chemicals and ionic bombardment inevitably cause numerous side-effects like hair loss, cardiotoxicity, infertility, chromosomal abnormalities and many more [4, 5]. Such life-inflicting consequences have strongly urged the development of patient-friendly therapeutic medicine including NPs-incorporated compounds.

Nanotechnology on the basis of peculiar material system, structures, shape and atomic stoichiometry at size scale below 100 nm yields unprecedented chemical, physical and biochemical properties enhanced by the phenomena of quantization and has already found pre-clinical and in vitro applications in many branches of biomedical science [6, 7]. Despite the drawbacks of chemotherapy, NPs serving as delivery carriers improve the selectivity of drug releasing in diseased tumor, facilitate drug uptake by the tumor cells and largely reduce the cumulative toxicity in the healthy tissue [8, 9]. Also, the image quality produced by an array of NP-based imaging modalities is greatly enhanced with higher sensitivity, finer spatial resolution and better depth penetration to reveal bio-distribution, monitor drug uptakes, localize tumor and evaluate efficacy of treatment [10]. In addition to the diagnostic function, the NPs when harnessed with inherent physical property, such as radiofrequency (RF)-ablation or photothermally rendered hyperthermia, can further induce damages on the desired location with enhanced efficiency [11–13], of which the second is commonly preferable over its site-specific dosing, lower degree of pain, low side-effects and much reduced risk of tissue burning.

Up-until-now, the NP material systems capable of inducing hyperthermia upon photo-irradiation include gold (Au), cesium tungstate (CsWO_3_), iron oxide, copper sulfide, graphene and carbon tube and demonstrated the applicability of imposing lethal damages on cancer cells by raising the extracellular or intracellular temperature in situ [14–18]. Alike the NP-enhanced RF-ablation, the level of incident power density of photonic sources is a critical issue for clinical safety and patient comfort [11], and it is manifested that the maximal exposure limit for human skin in the range of VIS and NIR wavelength between 400 to 980 nm is from 0.2 to 0.726 W/cm^2^ according to the International Commission on Non-Ionizing Radiation Protection (ICNIRP) published in 2013 [19]. Nonetheless, most of the optical power densities reported from the previous in vitro cancer cell studies were well beyond the safety limit for skin tissue, which can be a grave matter when it comes to treating internal biological tissues with a low threshold of photo-irradiation for vulnerability. For instances, the NIR optical power density of gold (Au) NPs that demonstrated effective destruction of cancer cells range from 2 to 80 W/cm^2^ when irradiated for no more than 10 min (min) [13, 14, 20–22]. Similarly, an assort of other material systems like graphene oxides [18], iron platinum (FePt) [23] and NaYF_4_:Yb,Er nanocrystals [24] required no less than 150 mW/cm^2^ for an instrumental deployment.

Being a relatively less explored material for in vitro experiment, a few studies reported annihilation of cervical cancer cells (Hela) by NIR-irradiated CsWO_3_ NPs with at least 0.72 W/cm^2^ [15, 25, 26] which is in the vicinity of skin tissue’s exposure limit of NIR wavelength set by ICNRP and may cause deleterious effects for healthy tissues under a prolonged duration of exposure [19]. Moreover, the temperature of culture media engendered by the combination of treatment dose of NP concentration, duration of photo-exposure and optical intensity, in the past studies, was well over 40 °C which is intolerable for healthy human cells, and the mortality rates of cancer cells were not delineated in great details.

CsWO_3_ NP is exceptionally absorptive in the range of NIR wavelength from 800 nm up to 2400 nm [27] and is functionally suitable for biomedical application. Despite its demonstrated outstanding efficacies in eliminating cancer cells, the cytotoxicity is still largely unknown and the provision of a dosing formula of NP concentration of low cytotoxicity, short duration of irradiation and optical power density within the limit of safety photo-exposure for skin tissue is still lacking.

Research study herein attempts to assess in vitro the feasibility of annihilating HepG2 hepatic cancer cells cultured in a petri dish with a diameter of 5.2 cm utilizing non-cytotoxic NP concentration and optical power density well within the photo-exposure limit of skin tissue while maintaining the temperature of cell culture media at normal human body temperature of 36.5 °C. In detail, Cs_0.33_WO_3_ NPs with an average feature size centered around 120 nm were synthesized using a sequence of redox, thermal annealing and wet-grinding processes, and characterized with its surface morphology, crystallinity, and optical and temporally photothermal properties. In addition, the photothermal effects of variable dose parameters, duration of irradiation, concentration of the NPs and optical power density of NIR irradiation operating at the central wavelength of 980 nm, on the survival rate of the HepG2 cancer cells, were examined and judged to devise a combination of safety treatment dose.

## Methods

In this research, 102nd-generation of HepG2 hepatic cancer cell line derived from human primary tumor was cultured as an experimental model to evaluate cytotoxicity imposed by the NIR-irradiated home-made Cs_0.33_WO_3_ NPs and assess the therapeutic efficacies of various thermal dose well within the safety limit for skin tissue exposure and at a non-toxic NP concentration.

### *Synthesis of Cs*_*0.33*_*WO*_***3***_*** NPs***

**Fig. 1:**
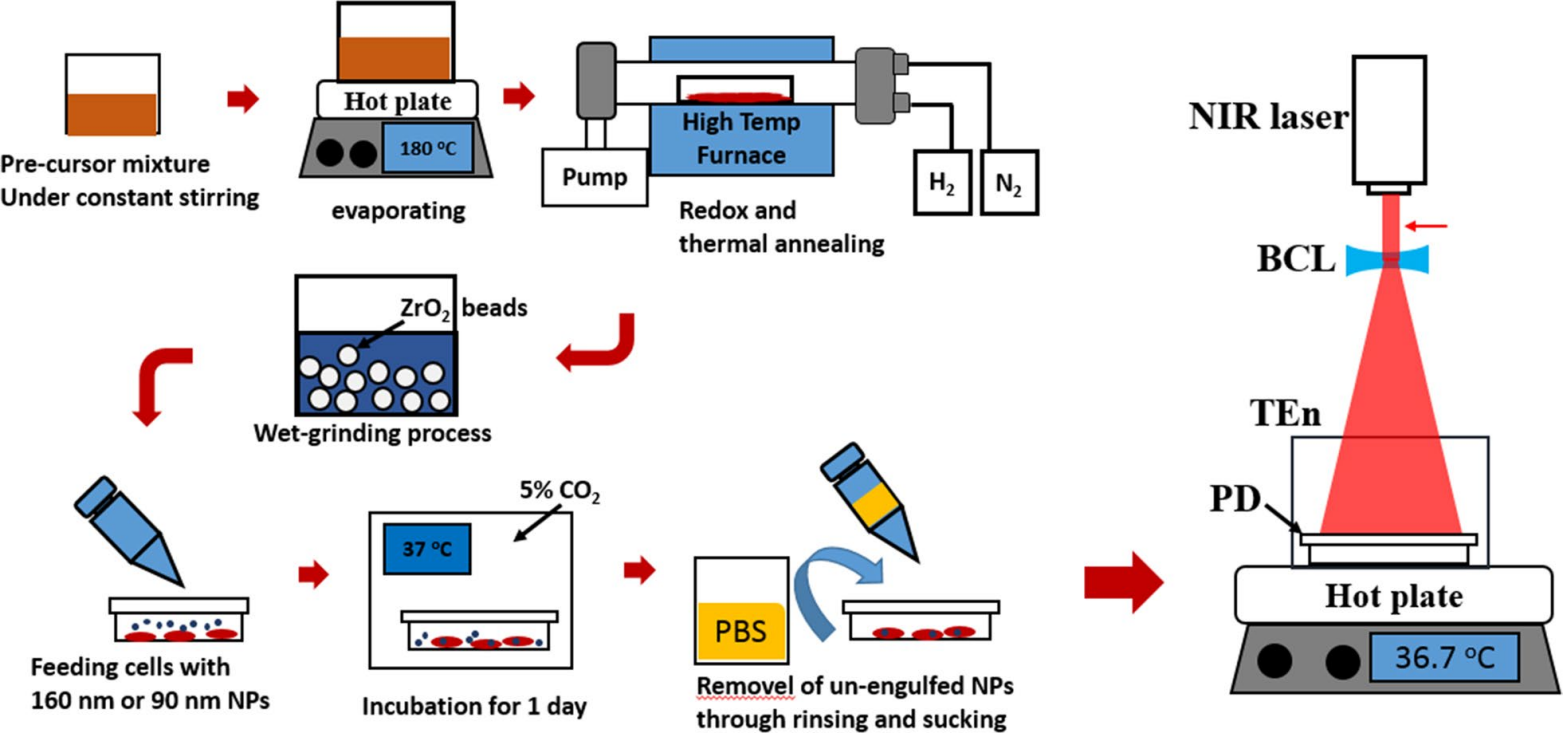
Schematic illustration of experimental procedures for material synthesis, cellular incubation with NPs and photothermal assay upon the cancer cells. BCL, TEn and PD are the abbreviation stood for bi-concave lens, thermal enclosure and petri dish, respectively; the red arrow indicates the location for the beam profile measurement

Left-hand panel of Fig. 1 illustrates the diagrammatic flowchart of the procedure of synthesis for cesium tungsten oxide (Cs_0.33_WO_3_) NP material. In brief, the precursor chemicals, ((NH_4_)_2_WO_4_) (Alfa Aesar, 99.9% purity) and CsCl (Alfa Aesar, 99.9% purity) were dissolved separately in 100 ml of DI water and then mixed together at 25 °C under a constant stirring at 250 round per minute (rpm) utilizing a magnetically actuated spinner for one hour (hr). After the stirring is done, the temperature of the mixture solution-containing beaker was adjusted to 180 °C and baked until the water content of the solution was completely evaporated. The resultant dried white powder was the final precursor of Cs_0.33_WO3 material. With the chiller turned on, the precursor powder-containing quartz boat was loaded into the center of a high-temperature furnace tube, and the pressure inside the furnace tube was brought to 0.08 torr. Afterward, the precursor is heated at 500 °C temperature alongside introduction of an inflow of a combination of gases, H_2_ and N_2_, in a ratio of 90 to 10 standard cubic centimeter per minute (SCCM) to facilitate redox reaction. After 1 h, the inlet of H_2_ gas is turned off, the flow of N_2_ gas is adjusted to 100 SCCM, and the temperature of furnace is raised to 800 °C for thermal annealing for one hour. After the processes were completed, the chiller and temperature-controlled furnace were turned off, and the quartz boat was cooled until it reached ambient temperature and was removed from the furnace tube. The resultant dark blue powder obtained from the quartz boat is the micron (µ)-size Cs_0.33_WO_3_ powder. To further scale down the feature size of the powder granules, 150 g of a mixture solution composed of 15 g of the µpowder, 3.8 g of a copolymer-based dispersant agent for preventing particles from aggregation, 10 μl of anti-foaming agent and D.I water was prepared, poured into a sample bowl that contained 600 g of zirconia beads and was mounted in the chamber of a nanogrinder equipment (Justnanotech Co., Taiwan). With the speed and temperature set to 2400 round per minute (RPM) and 15 °C, the NPs are produced by grinding the µpowder with 0.1 mm ZrO_2_ beads for 4 h, and with 0.05 mm ZrO_2_ beads for another 4 h. The total duration of each grinding process does not exceed 4 h to avoid excessive fluid viscosity as well as any erratic change of the physical sizes of the material. The final solution after the grinding process was sifted through a 0.22-μm pore filter for all subsequent characterization and experiment. The fluorescence version of Cs_0.33_WO_3_ NPs (fNPs) was made using the following protocol. A solution made of 2 ml of fluorescein at the concentration of 28 mg/ml and 2 ml of Cs_0.33_WO_3_ NP solution at 1.5 mg/ml was prepared in a beaker and placed in the bowl of an ultrasonic shaker for 15 min. Subsequently, the NP solution and the dispersant were mixed in a ratio of 1:1.25 and underwent an ultrasonic shaking for 15 min. The resultant solution was then washed with D.I. water and centrifuged at 10,000 rpm for 15 min. and repeated twice before any use.

### Material Characterization

Afterward, the characterization of Cs_0.33_WO_3_ NPs including NPs’ statistical feature sizes, crystalline structure, structural morphology, contour shape, VIS–NIR photo-absorbance was conducted using zeta potential analysis (ZS90, Malvern, UK), XRD (D2 Phaser, Bruker AXS GmbH, Germany), SEM (SU-5000, Hitachi, Japan) in conjunction with built-in energy-dispersive spectrometry (EDS), TEM (JEM-2100F, JEOL, Japan), dynamic light scattering (DLS) (Delsa Nano C, Beckman Coulter, U.S.A.), UV–VIS–NIR spectrometer (V-750, Jasco, Japan), respectively. The XRD spectra were acquired by scanning the X-ray upon the samples within an angular span from 20° to 80° at the scanning rate of 4° per min. The signal of scanning angle-dependent diffraction from the sample was determined and compared with the standard XRD spectrum of Cs_0.32_WO_3_ from the Joint Committee on Powder Diffraction Standards (JCPDS) card No. 83-1334. To confirm the temporal dependence of the NP's photothermal property, a simple experimental apparatus composed of NIR laser of 980 nm wavelength and a temperature measuring probe was set up to probe the state of temperature engendered by the NIR-irradiated solution. The examining solutions include the D.I. water-diluted NPs solution and the mixture of NPs solution in cell culturing media. The diameter of the optical beam for samples in the petri dishes was expanded to cover the entire surface of the petri dish, producing 0.05 W/cm^2^ in estimated optical power density, otherwise remained intact at 2 W/cm^2^. An optical setup shown on right-hand panel of Fig. 1 is utilized to perform NIR irradiation. At the heart of the optical system is a NIR laser beam directed toward a bi-concave lens that expands the beam diameter from 4 mm to 5.2 cm, an equivalent to the surface diameter of a petri dish placed on a hot plate set to 36.8 °C, which is a physiological temperature for cell growth; also, the petri dish is surrounded by a plastic cylindrical enclosure to help equilibrate the temperature of the ambient environment and the medium. Additional file 1: Fig. S1 illustrates the mapping of optical intensity of the NIR laser beam measured at the exit of the beam aperture which is indicated by a red arrow next to the laser beam in Fig. 1. The beam profile presents 3D distribution of optical intensity and verifies the uniformity of the light field over the entire opening of the petri dish.

### Cytotoxicity and Photothermal Assays

To begin the cycles of cell culturing, 500 ml of medium solution composed of 440 ml of Ham’s nutrient mixture F-12 and Dulbecco’s modified Eagle’s essential medium (HDMEM), 50 ml of fetal bovine serum (FBS), 5 ml of L-glutamine and 5 ml of P/S (Penicillin–Streptomycin), which was sterilized by a mesh filter with pore-size of 0.22 μm, was prepared. The cells-containing petri dishes, 10 cm or 5.2 cm in diameter, correspondingly filled with 8 ml and 2 ml of the medium were used for primary and sub-culturing and incubated in an incubator conditioned with 5% CO_2_ and at the temperature of 37 °°C. The observation of the cell growth and a renewal of the culture medium were done once every two days.

To obtain the survival rates for the cases of cell assays that include (1) control with no external input, (2) sole NIR irradiation, (3) incubation with NPs, and (4) incubation with NPs alongside an aftermath NIR irradiation, the culture medium in the culture dish was sucked out, and 0.4 ml of trypsin is added to the culture dish and placed in the incubator for about 10 min. Once detachment of cells from dish walls is confirmed, 10 μl of the cell-containing medium was drawn from the culture dish and added to 10 μl of trypan blue solution in a microcentrifuge tube, and a removal of the remnant floating NPs was done through several times of washing with phosphate buffer solution (PBS). Afterward, cell counting was carried out by filling a counting plate with 10 μl of the dyed cell solution through an injecting hole, and the cells can be observed at focal plane of a stereomicroscope and counted by a manual counter; each data point presented in all figures concerning cell survival rate was an average of three experimental trials (*N* = 3) plus the margin of standard deviation.

To prepare for the assessment of the NP’s cytotoxicity and its photothermal effects on the cellular survival rate, the medium in the 5.2 cm dish was removed, re-filled with proper amount of NP solution in the new medium to make an array of testing concentration, 2 mg/ml, 1.5 mg/ml, 1 mg/ml and 0.5 mg/ml, and then incubated for one day before the experiment.

Prior to the photothermal assay, the assessment of cytotoxicity was carried out to examine the response of the cells to an array of NP concentration and was done as follows. The medium containing the cells and NPs was taken out from the incubator and aspirated. One milliliter of pre-warmed PBS was used to wash the cancer cells and suck out remnant floating CsWO_3_ NPs that did not undergo endocytosis, the procedure of which is repeated several times to ensure that any potential cellular mortality is not caused by the NP-induced temperature rise in the new medium. After the photothermal treatment, the counting procedure was then implemented for the cytotoxicity and photothermal assay on the cells.

## Results

The optical absorbance and photothermal properties of Cs_0.33_WO_3_ nanomaterials are highly dependent on crystalline structure, post-annealing temperature, atomic stoichiometry and particulate feature sizes [28, 29].

**Fig. 2:**
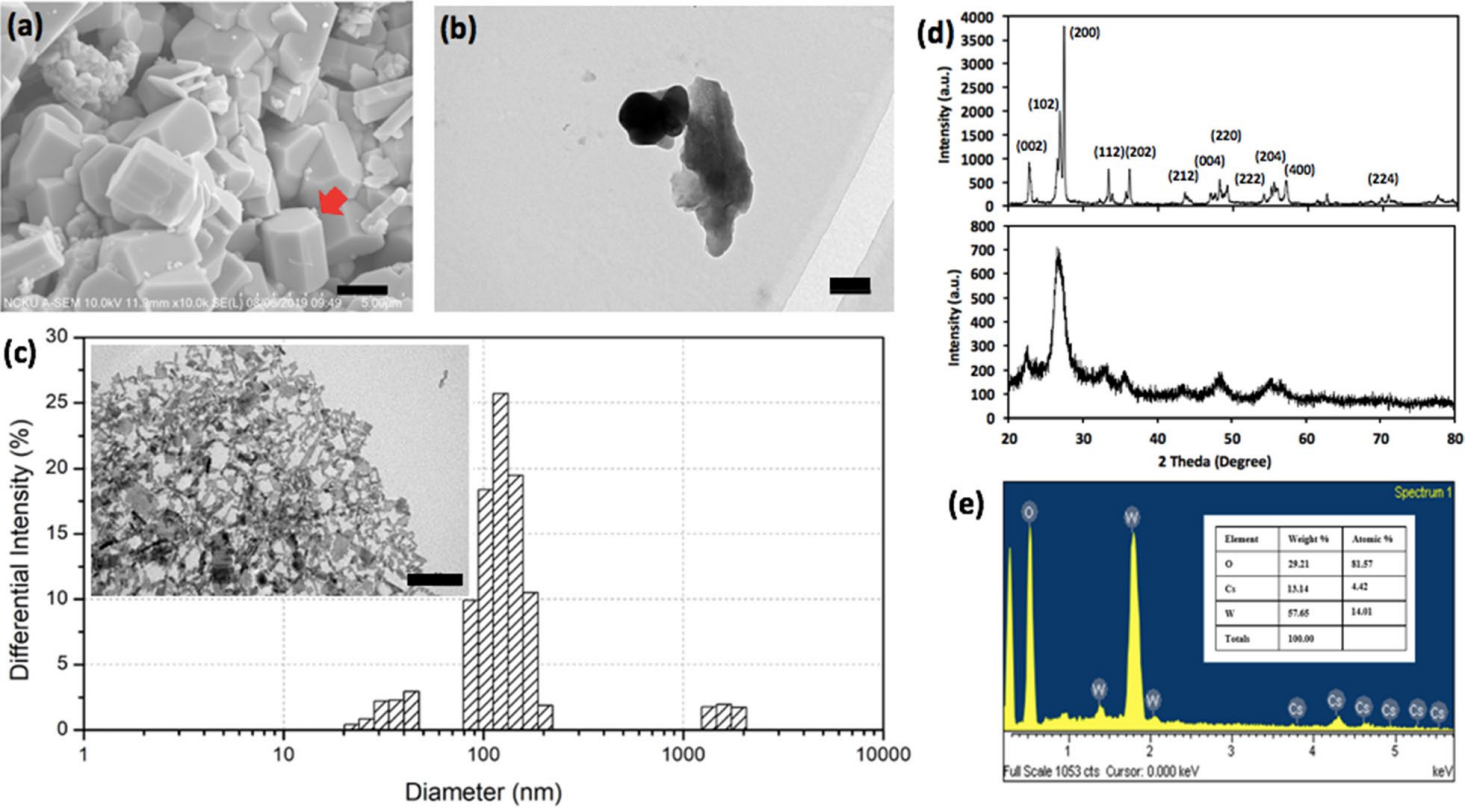
Physical and material characterization. **a** SEM and **b** TEM images of the µpowder, **c** DLS distribution histogram of NP feature size, **d** XRD spectra of µpowder and 120 nm NPs, and **e** EDS spectrum with percentage of atomic composition are presented. The scale bars in **a**, **b**, **c** are 1.5 μm, 200 nm and 100 nm, respectively

To characterize the surface morphology of the Cs_0.33_WO_3_ µpowder, SEM images with 10,000X magnification were acquired for visual confirmation of the iconic structure of columnar hexagon indicated by a red arrow in Fig. 2a. Additionally, the TEM images exhibit the contour shape and feature sizes of the µpowder granule in Fig. 2b where its feature size is around 1 μm or less. The NPs' rod-like geometry and the DLS distribution histogram of nanoscale feature size centered around 120 nm were also verified and are presented in Fig. 2c and the corresponding inset TEM image. Also, the crystalline characterization of the µpowder and NPs with XRD is presented in Fig. 2d. As can be observed from the µpowder's XRD spectrum on the top panel, the iconic planes of crystallization along (002), (102), (200), (112), (202), (212), (004), (220), (222), (204), (400) and (224) coincide well with the standard spectrum of Cs_0.32_WO_3_ from the Joint Committee on Powder Diffraction Standards (JCPDS) card No. 83-1334. When the feature size of µpowder reduces down to 120 nm, the intensities of all diffraction peaks are reduced monotonically, and some characteristic peaks indicating strong NIR absorption, such as planes (102) and (220), are dwarfed invisibly in the spectrum. Likewise, identification of the atomic constituents, cesium (Cs), tungsten (W) and oxygen (O), shown in Fig. 2e, not only confirms its atomic presence, but also authenticates the ratio of atomic percentages of Cs to W, 0.315, closely resembling the initially designed stoichiometry.

**Fig. 3:**
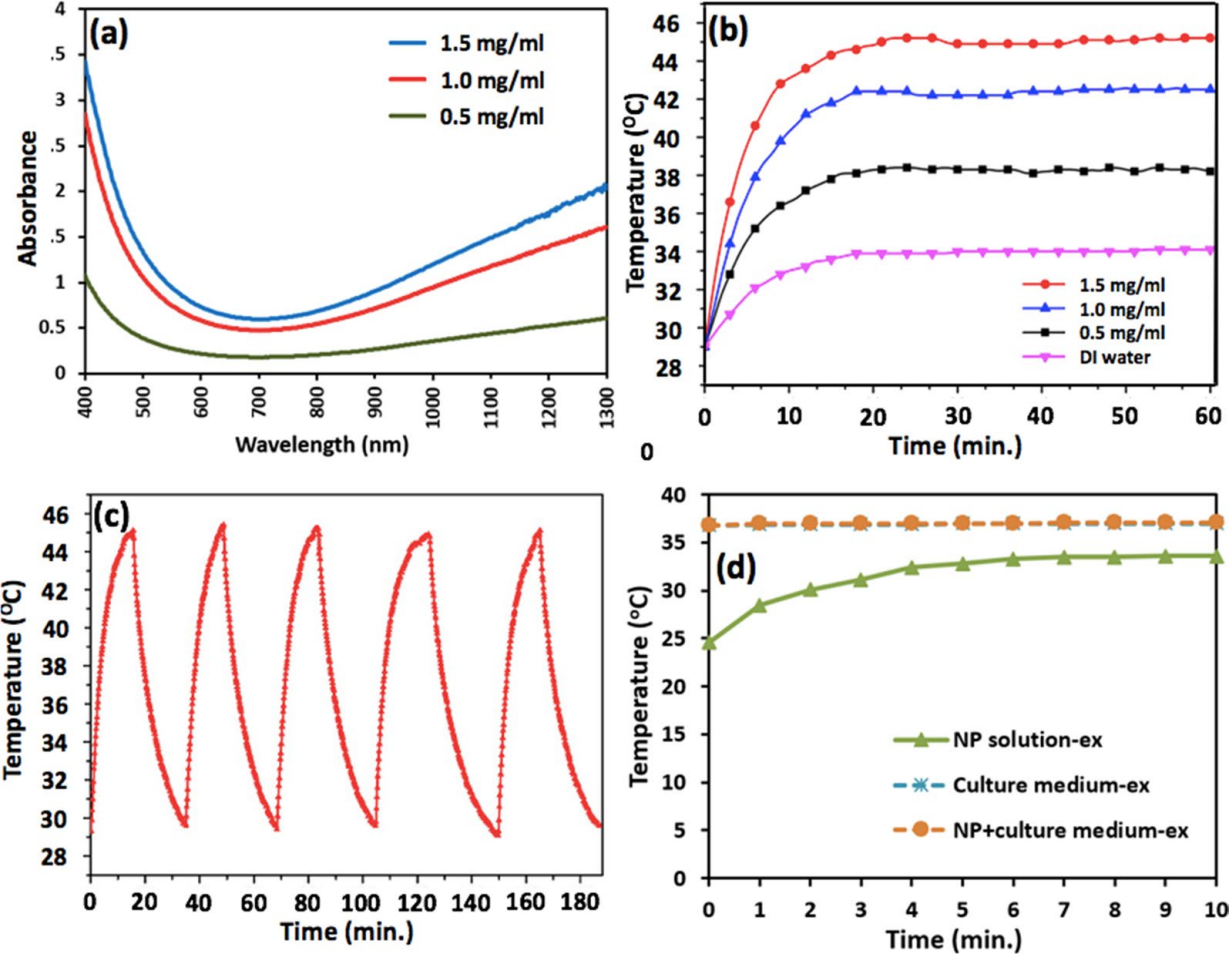
Optical and photothermal properties. **a** Optical absorbance spectra and the profiles of time-course temperature of NIR-irradiated NP solution in **b**, **c** a cuvette and in **d** a petri dish are depicted. Ex symbolizes the profiles of beam-expanded cases; NP concentration of 1.5 mg/ml and optical power density of 50 mW/cm^2^ were used in (**d**); the duration of NIR-irradiation per cycle in **c** is 15 min

Atop material characterization, the NPs' optical absorbance spectra and the time-course photothermal modulation in temperature were measured and are presented in Fig. 3. In (a, b), the dependence of NIR absorbance and the profiles of temperature rise induced by NIR-irradiated NP solution as a function of NP concentration are depicted, where the time-course temperature plot of 1 mg/ml, for instance, tops 40 °C, and remains steadily for at least 1 h confirming the materials' photothermal stability and durability. Likewise, the time-course temperature profile in Fig. 3c illustrates 5 repetitive cycles within 190 min, verifying the photothermal responsiveness of the NP material. In Fig. 3d, the temporal temperature profiles of the culture medium and NP-incorporated culture medium, taken out from the incubator, placed on the hotplate and undergone NIR irradiation, stabilize at around 37 °C over the course of 10 min., and the temperature of the NIR-irradiated pure NP solution rises from 24.6 °C up to 33.6 °C after 10 min. Thereby, in consideration of the NP's photothermal functionality, the NIR-irradiation for the following experiment was conducted for 10 min, 30 min and 60 min, maintaining robustness of the NPs during the experimental session and being potentially applicable to pre-clinical studies.

**Fig. 4:**
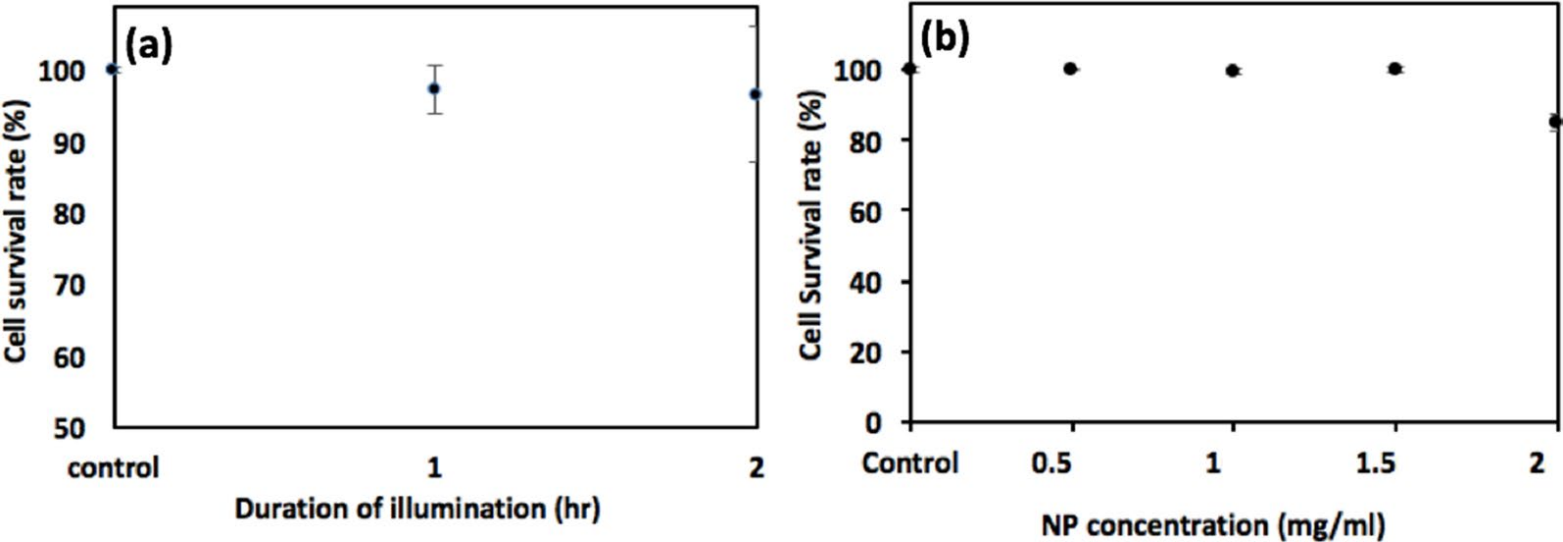
Cytotoxicity assay of experimental parameters. The survival rate of HepG2 cells when dosed with **a** the duration of NIR irradiation, **b** 120-nm NPs of various concentration are presented. The duration of NP incubation was one day; the standard deviation of three experimental trials (*N* = 3) for each data point is indicated

Subsequently, the non-toxic dose of experimental parameters including the duration of NIR irradiation and NP concentration was determined by irradiating the cells for 1 h and 2 h at 50 mW/cm^2^ and through direct interaction with the NPs of 0.5 mg/ml, 1 mg/ml, 1.5 mg/ml and 2 mg/ml in concentration, which are depicted in Fig. 4a, b, respectively. The cell survival rate remains well above 95% over the course of 2 h of NIR irradiation, confirming non-toxicity of the cells for a long-term exposure to the 980 nm photon, and also, the non-toxic NP concentration below 1.5 mg/ml was determined.

**Fig. 5:**
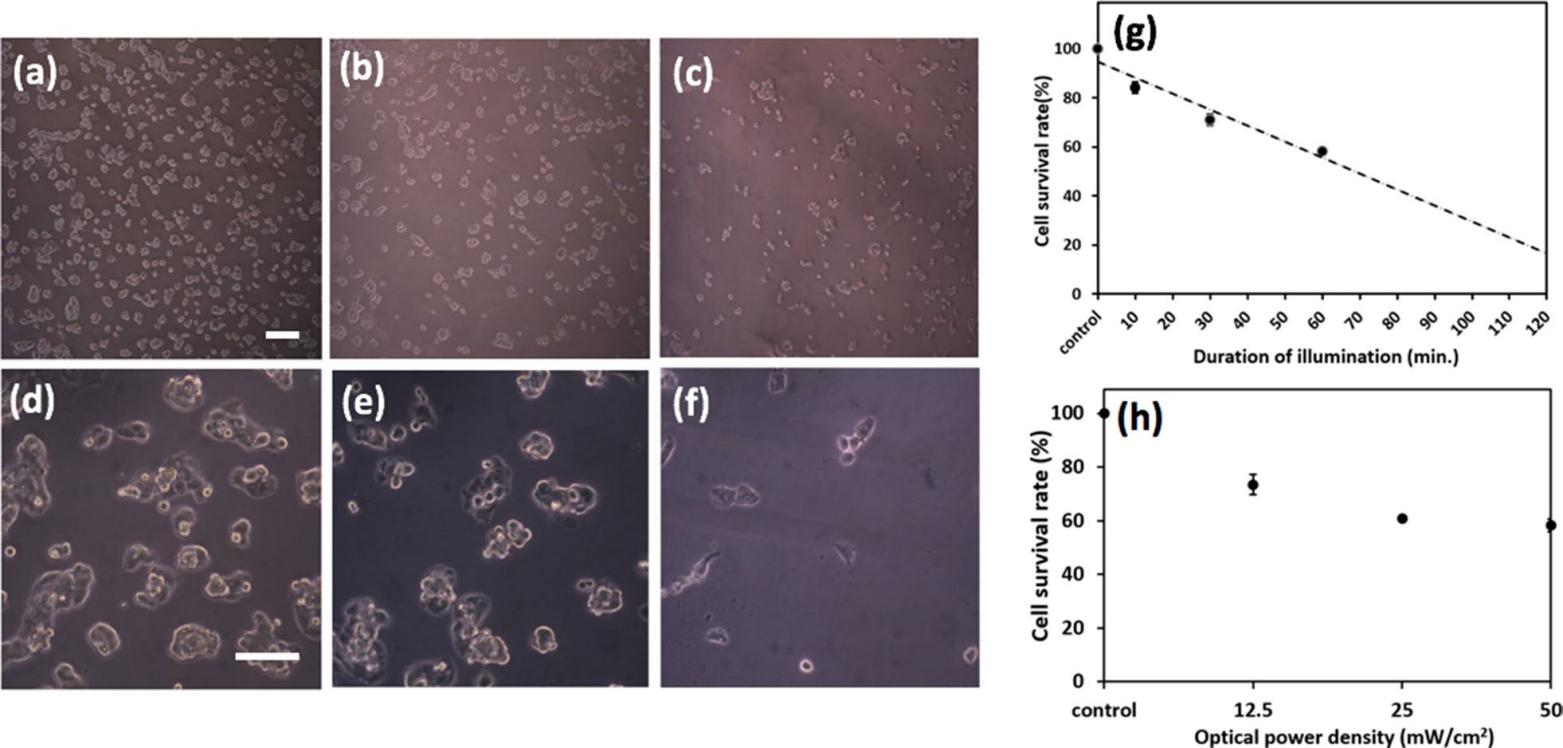
Photothermal assay. The survival rate of HepG2 cells when dosed with the NP concentration of 1.5 mg/ml alongside NIR irradiation. The respective scale bars on the **a**–**c** top and **d**–**f** bottom rows of BF optical images are 200 μm and 100 μm. The standard deviation of three experimental trials (*N* = 3) for each data point is indicated

The purpose of dosing the cancer cells at 1.5 mg/ml or lower, which has barely no damaging effect to HepG2 cells, is to examine the effects of photothermal dose on the cancer cells without the implication of the NP’s inherent toxicity. To examine whether the NIR-irradiated NPs can be a viable solution to eliminating cancer cells, the cells were incubated with NPs at 1.5 mg/ml for a day, and subsequently, imposed to the NIR radiation for 1 h. As can be seen from the bright-field (BF) optical images of Fig. 5d–f the number of cells is clearly reduced when the exposure time lasts 1 h (e) or 2 h (f). Quantitatively, the survival rate dwindles monotonically from 84.2% to 58.4% as the duration of irradiation increases from 10 min. to 1 h, and a linear trendy line fits well among the scattered data points, which predicts 20% of the survival rate when the irradiation lasts 2 h. In addition, Fig. 5h indicates that for 1 h of irradiation, the survival rate decreases from 73 to 58% as the optical power density increases from 12.5 to 50 mW/cm^2^, ascertaining the functionality of the NIR-irradiated NPs as a photothermal trigger in the destruction of cancer cells.

**Fig. 6:**
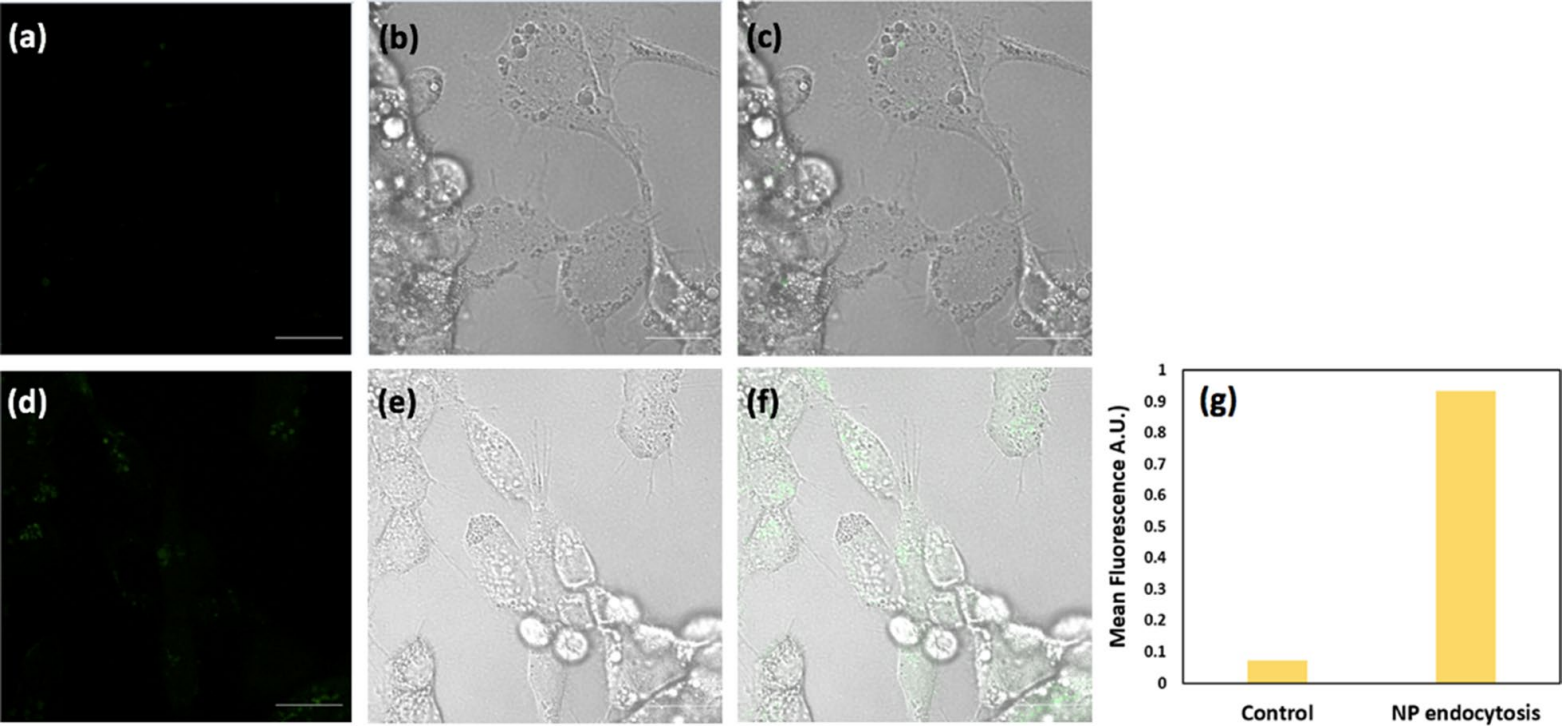
Optical confocal images. The **a**, **d** fluorescence, **b**, **e** BF and **c**, **f** composite optical images of the HepG2 cells incubated with and without the fNPs, as the corresponding experimental and control groups, are presented in the **a**–**c** top and **d**–**f** bottom rows alongside **g** a histogram of the average fluorescence intensities. Scale bars represent 20 μm; the laser excitation wavelength is 488 nm

Furthermore, the uncertainty over whether such photothermal action occurred with an intra-cellular or extracellular manner was addressed by preparing the fNPs, carrying out the same washing and incubation procedure, and observing any intracellular presence of the fNPs. Figure 6 illustrates confocal BF (b, e) and fluorescence (a, d) images and their superimposed composites (c, f) of the cells incubated with and without the fNPs. Evidently, the cells without incubation of fNPs as the control exhibit negligible green fluorescence, which is mainly attributed to the cellular autofluorescence, standing in sharp contrast to the experimental where the distribution of green fluorescence is omnipresent within all cytoplasm found in the image. The average fluorescence intensities in the images of control and experimental samples were also quantified and are presented in the histogram of Fig. 6g where the fluorescence of fNPs undergone endocytosis is at least ninefold as strong as the control.

## Discussion

The concept of therapeutic hyperthermia using electromagnetic waves in curing cancer diseases dated back as early as the early 1900s and was succeeded in retreating some forms of malignancies, but however, was waned due to the usefulness of fever-inducing antibacterial agents and the lacking of precise accessibility to local tumor of interests in situ [30]. Not until the 1980s, the interest was revived with several in vitro studies that discovered many aspects of metabolic changes, alteration of tumor microcirculation and acidolysis after hyperthermia treatment producing lethal effects on the cancer cells [31]. Mechanistically, besides direct cytotoxicity in which cancer cells undergo necrosis with a decreasing apoptosis when the applied temperature is > 42 °C, the reduced blood flow rate in association with lower cooling ability and low pH (< 6.8) render the cancer cells more susceptible to heating and consequentially, a higher cell-killing rate [32, 33]. However, clinically, due to the century-old drawback of imprecise non-specific localization, the hyperthermia only found enhanced therapeutic efficacy when applied concurrently with chemo- or radiation therapies [34, 35].

The NP-based materials that allow precise targeting and monitoring, and have a wide range of physical properties such as surface charge, fluorescence, photothermal conversion, fit nicely into the niche of such imprecision in hyperthermia application. However, despite the proven usefulness of many NIR-irradiated NP materials in cancer cell studies, the safety limit of photo-exposure is often not well examined as in the case of CsWO_3_ NPs. CsWO_3_ NPs, though, as shown in Fig. 4b, has relatively low cytotoxicity at 1.5 mg/ml when compared to a handful of popular NP materials, such as Ag, Au, graphene, whose thresholds lay on the scale of 1 μg/ml [18, 36, 37], still requires 0.7 W/cm^2^ for an effective destruction of Hela cells despite its strong NIR absorption in the wavelength range from 800 nm up to 2400 nm [15, 25, 26].

This research study intends to devise an effective NIR-irradiation-triggered, Cs_0.33_WO_3_ NP-based thermal dosing formula for in vitro HepG2 cancer cell as a function of NP concentration, duration of irradiation and optical power density well within the NIR exposure limit for skin tissue.

The experiment commences with the synthesis the NPs, where a step-by-step synthetic procedure including redox reaction, annealing process and wet-grinding method is outlined in Fig. 1. The redox reaction incorporates large ternary elements, Cs in this case, into rings of octahedral structure of WO_6_ in a proper physical environment, allowing formation of peculiar crystalline structure and incorporation of free electron into the metallic molecular compound, which is the intrinsic reason for strong photothermal conversion upon NIR absorption [26, 27]. Also, the subsequent annealing and wet-grinding processes helped refine the crystalline formation and reduce the particulate feature size that further enhance the NIR photothermal conversion. Afterward, the synthesized NP solution proceeded with a series of physical and material characterization that verifies an average feature size of 120 nm, a critical optimization of NIR absorption, and authenticates the atomic composition (Fig. 2). In addition, the corresponding measurement of zeta potential for 0.5 mg/ml, 1 mg/ml and 1.5 mg/ml are − 53.2 mV, − 54.3 mV, − 60.1 mV. Generally, the process of endocytosis is the main entry path for most of NP types, and the uptake rate of both cationic and ionic NPs for non-phagocytic cells is higher than neutral entities, though the former performing better than the later [38]. Also, many previous reports found negatively charged NPs less toxic to non-phagocytic cells [39, 40], which is a beneficial merit when an excessive intracellular NP accumulation for photothermal dose accompanied with a low toxicity is considered.

To set up a tone for the photothermal assay upon the HepG2 cells, the assessment of the NPs' robustness as a photothermal heater, its long-term stability at saturated temperature in equilibrium with the ambient environment over an hour and the repeatability for 5 consecutive cycles within 3 h were demonstrated (Fig. 3b, c). Also, to quantify the effectiveness of the NP's hyperthermia per se in dosing the cancer cells by ruling out the influence of ambient temperature (nominally at 25 °C), a calibration of the experimental apparatus was implemented by setting a hotplate to 37 °C, atop which all cell assays were carried out, and the temporally photothermal characteristics of the pure culture medium and NP-incorporated culture medium remained at 37.1 °C (Fig. 3d). Thereafter, the dependence of cytotoxicity on the duration of irradiation and NP concentration was examined separately and is presented in Fig. 4 indicating less than 5% of cell killing for over 2 h of NIR-irradiation and 1.5 mg/ml as the pivotal point toward lethal concentration. By fixing the NP concentration at 1.5 mg/ml, which was used throughout the rest of photothermal assay, the thermal dose for medical hyperthermia was defined as functions of variable dosing duration and optical power density. Figure 5 illustrates the action of cell killing with low (a–c) and high (d–f) magnification when NIR-irradiation for 0 h, 1 h and 2 h was implemented, the seemingly reduced number in the cells reflects well the quantitative analysis of cell survival rate as shown in (g), and the linear trend line predicts 80% of cell death for 2 h of irradiation. Likewise, the decrease in cell survival rate upon the incremental optical power density is also demonstrated in (h). Lastly, as clearly depicted by the BF, fluorescence and superimposed composite images in Fig. 6, in which a histogram of fluorescence analysis was presented, the endocytosis of the fNPs was verified.

## Conclusion

In summary, this study presents material synthesis and characterization of Cs_0.33_WO_3_ NPs, examines in vitro cytotoxicity assays of the direct NPs interaction, and separately, with NIR irradiation, and proves the endocytosis of the NPs as well as effectiveness of the NIR-irradiated NPs upon destructing the HepG2 cancer cells. Moreover, this study suggests a combinative dose of the NIR-irradiated Cs_0.33_WO_3_ NPs solution for the HepG2 cancer cells, 1.5 mg/ml of NP concentration, the duration of irradiation between 30 min. to 1 h, and optical power densities of NIR irradiation under 50 mW/cm^2^ which is well below the safety NIR exposure limit for skin tissue while allowing cancer cell mortality rate close to 40% and may be potentially applicable to the development of patient-friendly and personalized medicine. Such studies in a clinical setting will require additional measures like surface modification with molecules that recognize surface receptors of specific cancer cell types.

## Supplementary Information

**Additional file 1**. **Fig. S1.** Characterization of NIR laser beam. Illustration of **a** distribution of optical intensity on the surface front of the beam, **b** 3D depiction of the beam profile of optical intensity and intensity profiles along **c** Y and **d** X-axes are presented. Average optical power of 1W was used in this measurement.

## Data Availability

All data are fully available without any restriction.

## Abbreviations

NPs: Nanoparticles
fNPs: Fluorescence version of Cs_0.33_WO_3_ nanoparticles
NIR: Near-infrared
UV–VIS–NIR: Ultraviolet–visible–near-infrared
REDOX: Oxidation-reduction
DLS: Dynamic light scattering
XRD: X-ray diffraction
SEM: Scanning electron microscopy
EDS: Energy-dispersive X-ray spectroscopy
RF: Radiofrequency
ICNRP: International Commission on Non-Ionizing Radiation Protection
MIN: Minutes
SCCM: Standard cubic centimeter per minute
μ: Micron
RPM: Round per minute
JCPDS: Joint Committee on Powder Diffraction Standards
DI: Deionized
BF: Bright field
