# Supplementary figures and images for: Devising Hyperthermia Dose of NIR-Irradiated Cs0.33WO3 Nanoparticles for HepG2 Hepatic Cancer Cells
Source: Nanoscale Res Lett. 2021 Jun 26;16:108. doi: 10.1186/s11671-021-03565-4 (PMC8236016; doi:10.1186/s11671-021-03565-4)

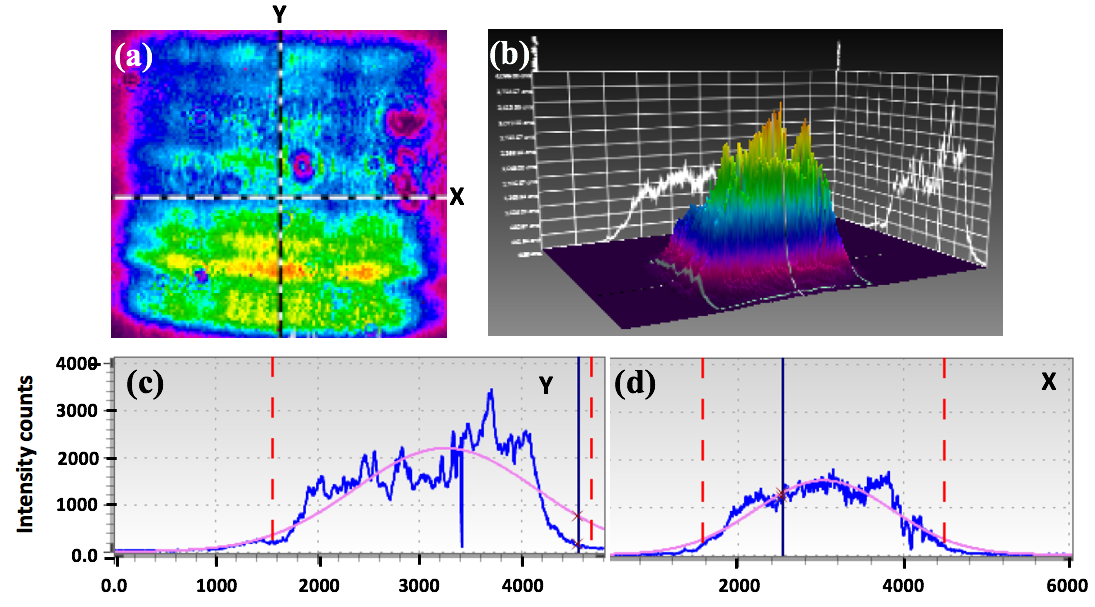

Supplement: Supplementary file 1 — Additional file 1. Fig. S1. Characterization of NIR laser beam. Illustration of a distribution of optical intensity on the surface front of the beam, b 3D depiction of the beam profile of optical intensity and intensity profiles along c Y and d X-axes are presented. Average optical power of 1W was used in this measurement. [file 11671_2021_3565_MOESM1_ESM.png]
